# Normative perceptual estimates for 91 healthy subjects age 60–75: impact of age, education, employment, physical exercise, alcohol, and video gaming

**DOI:** 10.3389/fpsyg.2014.01137

**Published:** 2014-10-07

**Authors:** Inge L. Wilms, Simon Nielsen

**Affiliations:** Brain Research and Advanced Technology Laboratory, Department of Psychology, University of CopenhagenCopenhagen, Denmark

**Keywords:** visual perception, normative estimates, processing speed, TVA, gaming, senior citizens, cognitive decline

## Abstract

Visual perception serves as the basis for much of the higher level cognitive processing as well as human activity in general. Here we present normative estimates for the following components of visual perception: the visual perceptual threshold, the visual short-term memory (VSTM) capacity and the visual perceptual encoding/decoding speed (processing speed) of VSTM based on an assessment of 91 healthy subjects aged 60–75. The estimates were modeled from input from a whole-report assessment based on a theory of visual attention. In addition to the estimates themselves, we present correlational data, and multiple regression analyses between the estimates and self-reported demographic data and lifestyle variables. The regression statistics suggest that education level, video gaming activity, and employment status may significantly impact the encoding/decoding speed of VTSM but not the capacity of VSTM nor the visual perceptual threshold. The estimates will be useful for future studies into the effects of various types of intervention and training on cognition in general and visual attention in particular.

## INTRODUCTION

The method demonstrated in this chapter is a computational assessment of visual processing capacity in 91 healthy subjects age 60–75. The output is a set of normative estimates as well as patterns of correlation between this capacity, demographic variables, and lifestyle variables as reference for future studies into the effect of training and intervention. The normative visual processing capacity estimates in this chapter is provided for the total sample of subjects as well as for the critical demographic variables: age, gender, level of education, employment status. In addition, we provide analysis on the influence of self-reported daily activities such as casual video gaming, alcohol consumption, smoking, physical exercise, and meditation.

### BACKGROUND

Many studies have demonstrated that the processing speed of the brain is susceptible to training throughout life (Takeuchi et al., 2011). This offers hope for prolonging the cognitive quality of life in both healthy and brain injured senior citizens through training intervention. However, cognitive effect studies are notoriously difficult to manage as many different aspects apart from the training itself may influence the cognitive ability being trained. In the recruitment of test subjects, it is therefore important to have normative data that reflects other activities which might influence the cognitive ability both before and during the training period.

As part of a larger study into the effects of cognitive training, we therefore wanted to try to model the visual capacity in a sample of healthy senior citizens taking into account the influence of demographic data as well as self-reported lifestyle activities. The idea was to try to assess which of the most common activities of daily living needs to be taken into account when doing cognitive effect studies within the field of visual attention.

The chapter introduces normative data collected from a whole-report paradigm based on a theory of visual attention (TVA; Bundesen, 1990). We chose the whole-report paradigm as it has been used for many years to measure the capacity of visual perception (Sperling, 1960; Rumelhart, 1970; Shiffrin and Gardner, 1972). Based on the whole-report data, TVA estimates the temporal threshold of conscious perception in milliseconds (*t_0_*), the speed of visual processing measured in letters per second (*C)* and visual short-term memory (VSTM) capacity measured in number of letters (*K)* of visual attention (Duncan et al., 1999). The advantage of using TVA-based assessment is the unspeeded accuracy-based measures which make it possible to characterize different aspects of attention avoiding confounding impact from motor components. This is particularly important when investigating effects of training or specific conditions (e.g., brain injury or neuropsychiatric disorders), which might affect both perceptual and motor functioning. TVA assessment has previously been used to successfully account for a range of behavioral and neurophysiological attentional effects (for a review see Bundesen and Habekost, 2008), and the theory provides a theoretical and empirical framework for investigating and explaining attention in both normal subjects (Finke et al., 2006; Jensen et al., 2012) and patients (Duncan et al., 1999; Bublak et al., 2006; Habekost and Rostrup, 2006, 2007; Habekost and Starrfelt, 2009; Redel et al., 2012; Starrfelt et al., 2013). The retest reliability of the TVA assessment of the *C* and the *K* parameter has also been demonstrated to be robust (Habekost and Bundesen, 2003; Habekost and Rostrup, 2006; Habekost et al., 2014). Different parameters can be modeled based on input from different paradigms. Other examples of paradigms for TVA assessment include the partial-report paradigm which also estimates the selectivity to perceiving targets in the presence of distractors (Vangkilde et al., 2011) and a single letter paradigm (Petersen and Andersen, 2012) which measure psychometrics of object identification in visual processing.

### ABOUT ASSESSMENT USING TVA ESTIMATES

Theory of visual attention is a formal computational theory for the way the visual attention system selects amongst incoming visual stimuli relevant for the task at hand (for a comprehensive account see Bundesen, 1990). According to TVA, the selection amongst incoming visual stimuli is a parallel processing race in which the attributes of objects in the visual field compete for access to a VSTM with a limited capacity of *K* elements. Only *K* number of items will, at any time, be selected and encoded into VSTM for later conscious actions. However, in line with the ideas of Desimone and Duncan (1995) the race is seen as a biased competition, in which the chances of winning the race are not equal for all objects and categories. Other aspects of the items in the visual field such as priming effects, spatial distribution, prior training, noise, and contrast may influence the probabilities of the encoding speed of certain objects and categories.

Encoding into VSTM is thought to proceed in two stages: in the first stage, attentional weights are computed and assigned to each element in the visual field according to their relevance. In the second stage, the total processing capacity of the visual system is distributed amongst the elements in proportion to their attentional weights. The capacity allocated to a particular element determines how fast this element is processed and how likely it is to become encoded into VSTM.

## METHODS

### THE WHOLE-REPORT ASSESSMENT

**FIGURE 1:**
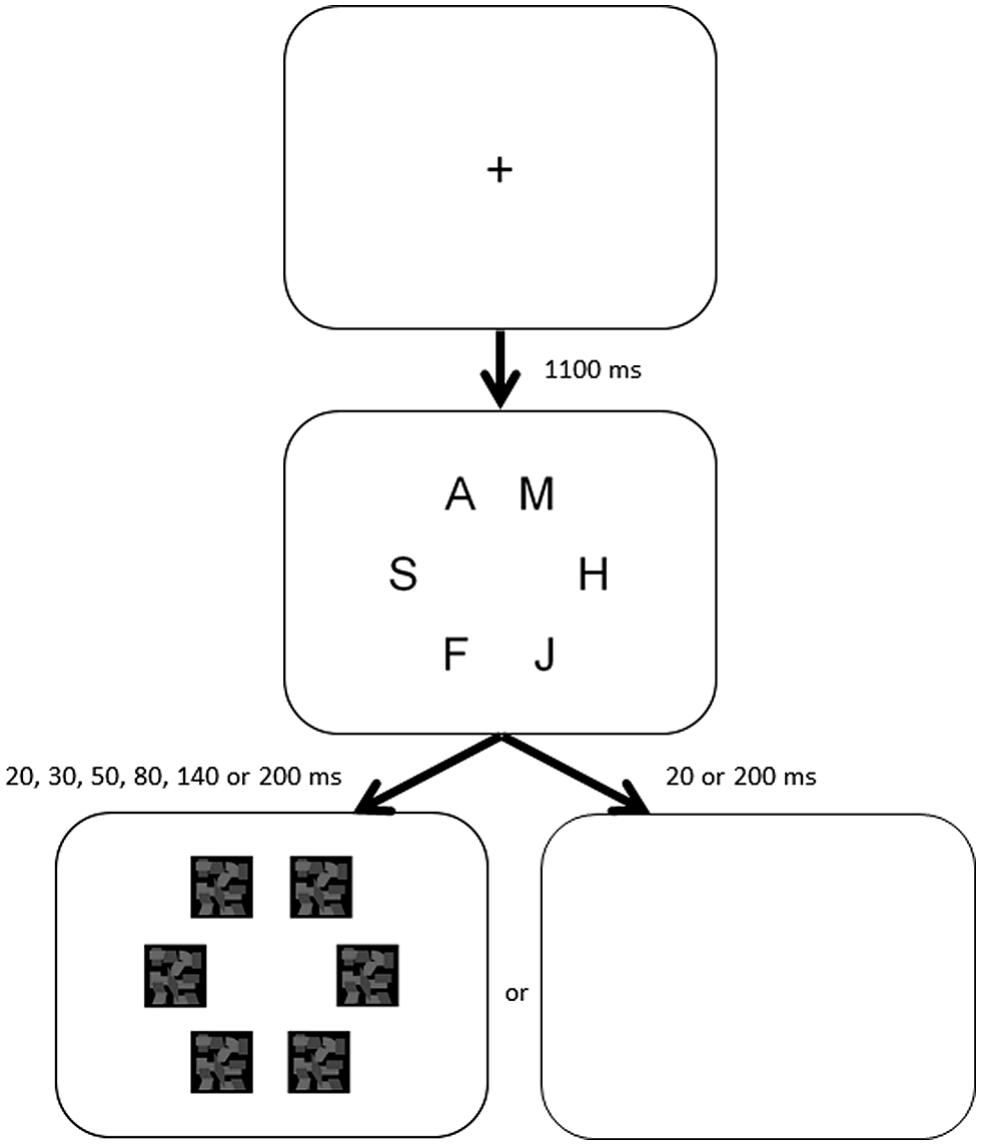
**Trial outline for the whole-report TVA test**.

A whole-report assessment was conducted using software developed in PsychoPy (Peirce, 2007), which captured the response of the test subjects. The sequence of the whole-report TVA paradigm is outlined in **Figure 1**. Each trial is initiated by pressing the space bar, which allowed the subjects to control the speed of progress throughout the assessment. Upon the initiation of a trial, the subjects were asked to fixate on a centrally placed cross. After 1100 ms, a stimulus display appears with six letters distributed on an imaginary circle at 9° eccentricity. Each stimulus subtended a visual angle of 2° at an approximate viewing distance of 65 cm. The stimulus duration was varied pseudo-randomly within blocks between 20, 30, 50, 80, 140, or 200 ms per trial. The trial exposure time was limited to 200 ms to avoid eye movements from confounding results. All 30, 50, 80, and 140 ms trials were immediately followed by a 500 ms mesh screen masking the positions of the six letters. The 20 and 200 ms trials were in some cases followed by the same mesh screen or in some cases a blank screen, the so-called unmasked condition. The masking conditions are included to ensure that estimates are made on the actual encoding and not a visual after-image. The unmasked conditions of the fastest and slowest exposure trials are included to improve modeling of the estimates of *t_0_* and *K*.

Subjects were encouraged to report the letters shown as best they could, aiming for an accuracy level between 70 and 90%. The trial procedure was self-paced by the subjects pressing the space bar. The test comprised eight blocks each of 36 trials. The first block was a training block. Thus seven blocks and a total of 252 trials with 28 repetitions per exposure duration were included in the analysis.

All tests were run on 21” ViewSonic G220f 215 CRT displays with a vertical refresh rate of 100 Hz to ensure precise timing of stimuli.

### PARTICIPANTS

A total of 91 healthy subjects were included in the test. The subjects aged 60–75 (*n*=91, *M* = 67.7, SD = 4.2), males (*n* = 28, *M* = 68.9, SD = 4.2) and females (*n* = 63, *M* = 67.1, SD = 4.1), with different educational backgrounds and employment status. The subjects were recruited through the Facebook page of the local branch of The DaneAge Association (“Ældresagen”), an interest group for senior citizens in Denmark and through advertisements in local newspapers. The participating subjects received a courtesy gift of chocolate and wine as well as thanks for participation. After signing a form of consent, potential participant were directed to a website where they were informed about the inclusion criteria. They had to be healthy with no history of brain injury, dementia, and diabetes. They would also be excluded if they currently were under medical treatment for psychiatric disorder or suffered from color blindness. Eyesight (self-reported) had to be normal or corrected to normal.

A total of 167 potential participants filled out an initial questionnaire about demographic data, activities of daily living and self-reported cognitive functioning. Based on the responses, 55 subjects were excluded either because they did not fulfill the inclusion criteria or because they had personal reasons not to continue. The remaining 112 participants were tested with the TVA whole-report paradigm and other cognitive assessments including MMSE. Data from a total of 21 subjects were excluded from the analysis, 7 due to modeling error, and 11 because they failed to comply with the task instructions. An additional three subjects were excluded from the normative material and analyses, because their TVA estimates fell beyond ±3 SD of the sample mean (Cousineau and Chartier, 2010). The exclusion of the three subjects had no consequence on the primary findings.

The randomness of the initial recruitment and subsequent exclusion of subjects resulted in a difference in the representation at the gender level (63 females, 28 males). This was taken into account in the further processing of data.

### THE QUESTIONNAIRE

As part of the initial recruitment, subjects were asked to fill out a detailed questionnaire regarding their daily activities and lifestyle. We asked about all the information that we anticipated might influence cognitive abilities like level of education, state of employment, state of health both physically and mentally, the use of drugs both legal and illegal, smoking habits, alcohol consumption, physical exercise habits, the use of social media, meditation, and video gaming. This was to allow an investigation into the possible influence of these factors on the perceptual system. The inclusion of questions regarding gaming habits in this population may seem odd at first but the Danish society is highly digitized to the extent that e-mail and internet-based service is the regular way of communication with public and private enterprise. Senior citizens have been encouraged to learn the use of computers and iPads and are regular users of both.

### STATISTICAL PROCEDURES AND MODELING

The whole-report data were fitted with the TVA framework (Bundesen, 1990) using MATLAB and the LibTVA toolbox (Dyrholm et al., 2011) to extract the *t_0_*, *C,* and *K* parameters. The LibTVA toolbox and user guide can freely be downloaded from http://zappa.psy.ku.dk/libtva.

All statistics were produced using IBM SPSS v.20. In addition to the normative statistics in Section “The Normative Estimates (**Table 1**),” we assessed the relation between the TVA parameters (dependent variables) and the demographic- and lifestyle variables (independent variables). To this end we used bivariate correlation procedures to estimate Pearson’s correlations coefficients, and multiple regression procedures to estimate the causal influence and strength of prediction of the independent variables, on each of the TVA estimates.

**Table 1:** Normative statistics of TVA estimates.

		*t_0_*			*C*			*K*		
		Mean	SD	*n*	Mean	SD	*n*	Mean	SD	*n*
Total for sample		22.76	16.14	91	44.05	17.95	91	3.53	0.72	91

Gender	Female	22.37	14.82	63	44.87	18.34	63	3.54	0.67	63
	Male	23.62	19.04	28	42.19	17.22	28	3.51	0.84	28

Age_Group	60–64	21.19	12.25	23	49.95	19.04	23	3.60	0.72	23
	65–69	23.29	14.89	36	44.38	18.83	36	3.50	0.71	36
	70–75	23.28	19.91	32	39.44	15.17	32	3.52	0.76	32

Retired	Yes	22.95	15.68	73	41.39	16.51	73	3.50	0.69	73
	No	21.95	18.35	18	54.84	19.93	18	3.67	0.84	18

Education_Group	1	25.83	19.70	15	36.51	17.68	15	3.40	0.85	15
	2	25.97	13.74	16	39.89	15.65	16	3.30	0.55	16
	3	19.74	14.38	38	49.49	18.01	38	3.74	0.70	38
	4	23.53	18.07	22	42.82	17.82	22	3.44	0.72	22

Gaming	No	21.39	17.01	49	39.31	17.44	49	3.49	0.70	49
	Yes	24.36	15.10	42	49.58	17.11	42	3.58	0.75	42

### ETHICAL CONSIDERATIONS

This project was registered at The Regional Danish Ethical Committee for research in Copenhagen and ruled to be a non-clinical trial. Written, informed consent was obtained from all participants.

## RESULTS

Normative data for TVA estimates will be presented for the demographic variables Gender, Retired, Age_Group, Education_Group, and Gaming habits. Following the normative data, correlation statistics are presented to illustrate the intra-correlations of the independent variables and of the TVA estimates. The relationship and influence of the demographic and lifestyle variables (alcohol, exercise, and casual video-gaming) on the TVA estimates will be analyzed using multiple regression statistics.

Due to the randomness of the initial recruitment and subsequent exclusion of subjects, the gender level differed substantially in size yielding a strong bias toward female (63 females, 28 males). To assess female bias on the TVA estimates, we ran a multivariate analysis of covariance with gender as fixed factor, the TVA estimates as dependent variables, and the demographic- and lifestyle variables as covariates, which showed no significant interaction of gender (all *p*-values > 0.37). Thus gender is assumed not to interact with the TVA estimates, for the demographic and life style variables presented here.

### NORMATIVE ESTIMATES

In **Table 1**, distribution measures of TVA estimates are presented for each of the demographic variables Gender, Age_Group, Retired, Education_Group, and Gaming.

Six categories of education were originally represented in the data [1 = elementary education (7–9 years), 2 = technical school, 3 = high school, 4 = 2 years of higher education in addition to high school, 5 = bachelor, 6 = master level, or higher]. These categories have been collapsed into four groups due to a sparse representation of subjects at the lower education levels. Group 1 comprises the three basic categories of education (1–3), Group 2 correspond to category 4, Group 3 to category 5, and Group 4 to category 6.

### INFLUENCE OF DEMOGRAPHIC AND LIFESTYLE VARIABLES ON TVA ESTIMATES

**Table 1** indicates that the TVA estimates vary depending on the grouping of data. In **Table 2**, correlational measures (Pearson) between the TVA estimates and the measured variables are presented. This includes demographic variables (gender, retirement status, age, level of education) and the lifestyle variables: alcohol consumption (A_DPM; Drinks per month, *M* = 26.4, SD = 23.4), physical exercise (E_HPM; Hours per month, *M* = 18.1, SD = 16.4), casual video-gaming (G_HPW; Hours per week, *M* = 1.5, SD = 2.4).

**Table 2:** Correlational measures between demographic and lifestyle variables, and the TVA estimates.

		*t_0_*	*C*	*K*	Gender	Age_Group	Retired	Education_Group	A_DPM	E_HPM	G_HPW
*t_0_*	*r*	1	0.078	-0.376	0.036	0.047	-0.025	-0.086	0.084	-0.176	0.210
	*p*		0.460	0.000**	0.736	0.659	0.815	0.416	0.429	0.095	0.046*
*C*	*r*	0.078	1	0.258	–0.069	–0.226	0.300	0.163	–0.003	–0.206	0.224
	*p*	0.460		0.013*	0.514	0.031*	0.004**	0.123	0.978	0.050*	0.033*
*K*	*r*	–0.376	0.258	1	–0.020	–0.039	0.097	0.086	0.020	–0.145	0.031
	*p*	0.000**	0.013*		0.853	0.716	0.362	0.419	0.853	0.170	0.770
Gender	*r*	0.036	–0.069	–0.020	1	0.162	–0.032	0.246	0.332	0.056	–0.073
	*p*	0.736	0.514	0.853		0.126	0.762	0.019*	0.001**	0.596	0.492
Age_Group	*r*	0.047	–0.226	–0.039	0.162	1	–0.350	0.034	0.155	0.227	–0.127
	*p*	0.659	0.031*	0.716	0.126		0.001**	0.751	0.143	0.030*	0.230
Retired	*r*	–0.025	0.300	0.097	–0.032	–0.350	1	–0.062	–0.123	0.008	–0.032
	*p*	0.815	0.004**	0.362	0.762	0.001**		0.560	0.246	0.939	0.761
Education_Group	*r*	–0.086	0.163	0.086	0.246	0.034	–0.062	1	0.060	–0.055	–0.183
	*p*	0.416	0.123	0.419	0.019*	0.751	0.560		0.570	0.603	0.083
A_DPM	*r*	0.084	–0.003	0.020	0.332	0.155	–0.123	0.060	1	–0.042	0.092
	*p*	0.429	0.978	0.853	0.001**	0.143	0.246	0.570		0.690	0.386
E_HPM	*r*	–0.176	–0.206	–0.145	0.056	0.227	0.008	–0.055	–0.042	1	–0.234
	*p*	0.095	0.050*	0.170	0.596	0.030*	0.939	0.603	0.690		0.025*
G_HPW	*r*	0.210	0.224	0.031	–0.073	–0.127	–0.032	–0.183	0.092	–0.234	1
	*p*	0.046*	0.033*	0.770	0.492	0.230	0.761	0.083	0.386	0.025*	

Significant correlations at the 0.05 level are marked with a * and highly significant correlations below the 0.01 level are indicated by **.

#### TVA intra-correlations

There was a positive correlation between *C* and *K*, which has been reported previously (Habekost et al., 2014). In addition, there was a negative correlation between *t_0_* and *K*, which to our knowledge has not previously been reported. We suggest that the interaction may be causally related to age, which is supported by previous studies reporting an age related increase in *t_0_* and decrease in *K* (McAvinue et al., 2012; Habekost et al., 2013). However, we were not able to confirm this hypothesis in our data (see Discussion of Effects of Age).

#### Variables correlating with TVA estimates

There was a significant positive correlation (*p* < 0.05) between the processing speed estimate *C* and Age_Group and G_HPW (gaming hours per week). Surprisingly, there was a significant but negative correlation (*p* < 0.05) between *C* and E_HPM (physical exercise hours per month; see Discussion of Effects of Physical Exercise). There was a highly significant correlation (*p* < 0.01) between the processing speed estimate *C* and retirement status (Retired).

While **Table 2** suggests interaction between some of the demographic and lifestyle variables with the TVA estimates, the correlational measures are prone to confounding biases from other factors. So to assess the causal relation and individual contribution of variance of the demographic and lifestyle variables (independent) to the TVA estimates (dependent), multiple regression analyses were run for each of the TVA estimates. The assumptions of linearity, independence of errors, homoscedasticity, unusual points and normality of residuals are all met for the analyses. Furthermore, there were no multicollinearity issues between the independent variables (which can also be verified from **Table 2**: all *r* < 0.7).

The analysis showed that the demographic and life style variables did not predict the variations in visual perceptual threshold (*t0)*, *F* (7,83) = 1.04, *p* = 0.41, adj. *R^2^* = 0.003, *p_min* > 0.13. Nor did they predict the capacity of VSTM (*K)*, *F* (7,83) = 0.51, *p* = 0.83, adj. *R^2^* = -0.04, *p_min* > 0.12 – where *p_min* corresponds to the significance level of the most influential independent variable. This means that when controling for the covariance in the reported measurements, no demographic or lifestyle variables significantly influenced the perceptual threshold *t_0_* or the VTSM capacity *K*. However, the demographic and lifestyle variables did predict the *C* parameter, *F* (7,83) = 3.49, *p* = 0.003, adj. *R^2^* = 0.162, and three of the variables contributed significantly (Retired, Education_Group and G_HPW). The results from the Multiple Regression analysis can be found in **Table 3**.

**Table 3:** The influence of lifestyle and demographic variables on the perceptual processing speed estimate *C.*

	*B*	SE_B_	β	*t*	*p*
Intercept	22.945	11.079		2.071	0.041
Retired	13.592	4.671	0.303**	2.910	0.005
Age_Group	–1.414	2.512	–0.061	–0.563	0.575
Gender	–3.758	4.122	–0.097	–0.912	0.365
Education_Group	4.293	1.813	0.241*	2.367	0.020
A_DPM	0.027	0.080	0.035	0.337	0.737
E_HPM	–0.132	0.112	–0.120	–1.173	0.244
G_HPW	1.760	0.779	0.231*	2.257	0.027


B = unstandardized regression coefficient; SE_B_ = standard error of coefficient; β = standardized coefficient. The regression coefficients describe the slope of the linear relation between the specific input variable, and the output variable when the other input variables are fixed. Significant predictors at p < 0.01 level is marked with ** and at *p* < 0.05 level is marked with *.

## DISCUSSION

### THE NORMATIVE ESTIMATES (TABLE 1)

The way the sample was recruited created a potential bias when compared to the performance of the general aging population and require a word of caution. Firstly, it takes a certain amount of motivation and interest in science and research to act upon a written or electronic request to join a research program. Secondly, the level of education in the sample used is not entirely representative of the Danish population in general. According to information from the Statistics Denmark database, the current general level of education amongst the target population aged 60–69 is as follows: ∼30% of the population has only completed elementary school, 2% has completed high-school, 42% has a technical or craftsmanship education, 4% a short education above high school, 16% had a bachelor education and 6% a master’s degree or higher.

In our sample, only 5% had completed elementary school only, almost 40% had a bachelor education and 20% had a master’s degree or higher. So it might be a valid concern for the normative estimates that our sample is skewed toward a higher educational level. We chose to collapse the three basic levels of education (7–12 years of education) into one category (1) to improve the power of the measures. When using the estimates, it should be taken into consideration that category 1 includes participants with more than just the basic educational requirement of 9 years.

Thirdly, the sample was screened for serious illness and medication that would impact cognitive training before entering the trials.

### EFFECTS OF EMPLOYMENT STATUS

Retirement from work seems to be linked to a reduction in the estimates for perceptual processing speed *C*. Those participants, who were still employed part time or full-time, performed better in the TVA assessment than the retired participants.

This was a surprising finding as we had expected no influence from employment state. A valid argument could be that the retirement group is older than the employed group. However, as can be seen in **Table 3**, retirement status contributed significantly to the variation in perceptual processing speed even when adjusting for age, indicating that those still employed have a higher perceptual processing speed (**Table 1**: *C* = 54.84) than those retired (**Table 1**: *C* = 41.39). There may be several explanations for this difference. It is well known that social engagement stimulates cognitive ability (e.g., Bassuk et al., 1999; Glei et al., 2005). Retirement from work will in many cases result in reduced social interaction and over time perhaps contribute to cognitive decline. Also, mental stimulation has been shown to be a preserver of cognitive functioning in old age (Wilson et al., 2012). The variety and demands from the working environment itself may very well contribute to the stimulation of abilities such as attention and memory. That our sample was strongly biased toward the highly educated, may further support this notion since the cognitive demands imposed by positions employed by highly educated are likely to be greater than those employed by people with lower levels of educations. Even navigating through traffic to and from work may play a role particularly on attention. In a study from Anguera et al. (2013) demonstrated that there was room for improvement of abilities like cognitive control in the 60–85 year olds.

In summary, the findings encourage considering controling for employment status when recruiting a homogeneous sample for future cognitive effect studies.

### EFFECTS OF EDUCATION

Level of education seems to have a positive effect on perceptual processing speed. Other studies from France, Mexico, and USA have investigated the relationship between level of education and cognitive preservation on larger sets of the populations (Ardila et al., 2000; Alley et al., 2007; Glymour et al., 2012). The findings were inconclusive at the general cognitive domain of attention. In terms of cognitive assessment and education, a positive relationship has been demonstrated between level of education and cognitive performance (Glymour et al., 2012).

One explanation might be that higher education tends to imply highly developed reading ability. Reading is known to affect visual processing speed (Jackson and McClelland, 1979) and so it may be reading proficiency more than education *per se* that makes a difference. As we did not ask people in details about their reading habits, this requires further investigation.

### EFFECTS OF VIDEO GAMING

In Green and Bavelier (2003) demonstrated that young people playing action video games showed improved visual selective attention. The effect was attainable through training and not a result of some innate superior ability. Since then many studies have demonstrated superior visual ability in video gamers in areas like spatial resolution (Green and Bavelier, 2007), temporal auditory and visual sequencing of external stimuli (Donohue et al., 2010) and task switching (Karle et al., 2010). Further investigations into the specific elements of attention suggest that the improvements are facilitated by faster encoding and decoding to VSTM (Wilms et al., 2013). It therefore seemed prudent to include questions about gaming habits when trying to establish normative estimates for visual perception. In the study, we specifically asked the participating subjects about their video gaming habits, the frequency and duration of playing as well as details about the type of games played if any.

Forty-two subjects responded positively to playing video games on either iPad or PC. When asked about the type of games played, most subjects answered “Brainteasers” like Sudoku, Tetris, Candy Crush, Angry Birds, and Wordfeud etc. and many supplemented this with online card games like Solitaire, Poker, and Bridge.

Casual video gaming was a significant predictor of *C*, such that the number of hours spent playing video games was positively related to the perceptual processing speed. The results are surprising as many would argue that apps like Candy Crush do not fall into the standard category of action video games.

However, we speculate that in terms of gaming impact, it may not be only the action of the game that place demand on the perceptual system. Many of the relaxation apps have an element of time limitation, which requires the gamer to respond to a challenge within a given time frame. We speculate if this may in fact be the reason why we see an effect of frequent gaming. It certainly raises the question whether other types of modern video games not normally categorized as action games may have a positive impact on cognition.

Since this is not a training study, we have no way of knowing of the difference in perceptual processing speed in gamers and non-gamers was innate, but previous studies support that gaming influences processing speed and our measures indicate that gaming needs to be controlled in studies of intervention.

### EFFECTS OF AGE

It has been the general consensus that perceptual ability decline over time (e.g., Kail and Salthouse, 1994) and that it may be related to decline in cognitive processing speed (Salthouse, 1996; Finkel et al., 2007). For that reason we had expected to find a general decline on all the perceptual estimates.

The initial correlation analysis (see **Table 2**) seems to indicate a relationship between Age_Group and C. However, when performing multiple regression analyses (**Table 3**) Age_Group does not contribute significantly to the variance in *C*. This means that within the age range of our sample, age does not influence the *C* value when controling for the influence of variables like education level, retirement status, and gaming habits. The same is true for *t_0_* and *K*.

This contradicts the findings in previous TVA studies (McAvinue et al., 2012; Habekost et al., 2013) which found a significant effect of age on the capacity of VSTM (*K*), the visual perceptual threshold (*t_0_*), and processing speed (C).

In Habekost et al. (2013), a significant decline was found in a slightly older population (aged 70–85, *n* = 33) and McAvinue et al. (2012) found a strong effect of age on processing speed (C). We speculate that the difference in findings may be due to the sample sizes in both studies being smaller than in our study. In addition, the individual age groups in those studies were including older age-groups. This would correspond to the findings by Rabbitt et al. (2001) that the decline from age 49–70 is much smaller than from age 70 and upward.

In terms of the result deviating from general observations we have to point out that our sample represents a highly functional and healthy group of people. General health plays an important role in preservation of cognitive ability (Wilson et al., 2012). Thirdly, the TVA assessment is unspeeded. Although the trials themselves are temporally limited by the exposure time, the response required from the subject was unspeeded. This may have reduced the cognitive load enough to counter effects of age-related decline normally found in general aging studies (Salthouse, 2000).

### EFFECTS OF ALCOHOL

Many studies have investigated the influence of drinking on cognition in the elderly population (for a review see Peters et al., 2008). The general consensus is that low to moderate consumption may have a positive and protective effect on cognitive functioning as we grow older.

We asked the subjects to estimate how often they drank alcoholic drinks and the amount consumed at each event. The data show an increase in alcohol consumption over age but did not demonstrate any significant impact of consumption on the estimates of perceptual processing speed, capacity of VSTM or the visual threshold. This supports that low to medium consumption of alcohol in our sample did not influence the TVA estimates.

### EFFECTS OF PHYSICAL EXERCISE

Physical exercise generally considered to be of benefit to the mental health in senior citizens (e.g., Clarkson-Smith and Hartley, 1989; Hillman et al., 2008). We asked the subjects to report the frequency and duration of physical training as well as the type of physical training practiced (if any). The data did not support that physical exercise influences the TVA estimates. There was a significant correlation between *C* and exercise (see **Table 2**) but further analyses showed that this was due to a confounder bias arising from a causal relation between Age_Group and *C* and Age-Group and physical exercise. The multiple regression analysis controls for these co-variance factors and the result was no relationship between physical exercise and processing speed (*C*; **Table 3**).

## CONCLUDING REMARKS

We presented the TVA estimates for a sample of 91 healthy subject aged 60–75 and influences on the estimates of self-reported physical exercise, alcohol consumption, video gaming as well as various demographic categorizations. We did this to provide a set of normative estimates to be used in future studies into effects of training and intervention as well as assessment of visual perception in relation to clinical conditions.

We found a significant effect of retirement status, gaming habits, and education level on the estimates of the perceptual processing speed as it relates to encoding of letters per minute.

Many studies of the capacity of visual attention have been done on younger, healthy student samples, which may not be representative of the population as a whole (e.g., Petersen and Andersen, 2012; Lohmann et al., 2013; Vangkilde et al., 2013; Wilms et al., 2013). It is our hope that the TVA paradigms in time will develop into a standard set of assessment tools for the basic elements of visual attention and that normative data will become available for other samples of the population.

In addition, studies into the effect of cognitive training have been conducted for many years and with inconclusive results. The data presented here supports the notion that in order to ascertain the effect of cognitive training, activities of daily living needs to be controlled for to avoid confounding the primary measurements. We need to improve and expand the availability of normative data for cognitive abilities susceptible to training both in healthy and injured populations through further research.

## Conflict of Interest Statement

The authors declare that the research was conducted in the absence of any commercial or financial relationships that could be construed as a potential conflict of interest.
